# Crystal Structure of Human TWEAK in Complex with the Fab Fragment of a Neutralizing Antibody Reveals Insights into Receptor Binding

**DOI:** 10.1371/journal.pone.0062697

**Published:** 2013-05-08

**Authors:** Alfred Lammens, Monika Baehner, Ulrich Kohnert, Jens Niewoehner, Leopold von Proff, Michael Schraeml, Katja Lammens, Karl-Peter Hopfner

**Affiliations:** 1 Center for Integrated Protein Science (CIPSM), Munich, Germany; 2 Department of Biochemistry at the Gene Center, Ludwig-Maximilians University, Munich, Germany; 3 Biologics Research, Roche Pharma Research and Early Development (pRED), Penzberg, Germany; 4 Research and Development, Roche Professional Diagnostics, Penzberg, Germany; University of Washington, United States of America

## Abstract

The tumor necrosis factor-like weak inducer of apoptosis (TWEAK) is a multifunctional cytokine playing a key role in tissue regeneration and remodeling. Dysregulation of TWEAK signaling is involved in various pathological processes like autoimmune diseases and cancer. The unique interaction with its cognate receptor Fn14 makes both ligand and receptor promising targets for novel therapeutics. To gain insights into this important signaling pathway, we determined the structure of soluble human TWEAK in complex with the Fab fragment of an antibody selected for inhibition of receptor binding. In the crystallized complex TWEAK is bound by three Fab fragments of the neutralizing antibody. Homology modeling shows that Fab binding overlaps with the putative Fn14 binding site of TWEAK. Docking of the Fn14 cysteine rich domain (CRD) to that site generates a highly complementary interface with perfectly opposing charged and hydrophobic residues. Taken together the presented structure provides new insights into the biology of TWEAK and the TWEAK/Fn14 pathway, which will help to optimize the therapeutic strategy for treatment of related cancer types and autoimmune diseases.

## Introduction

The TNF-like weak inducer of apoptosis (TWEAK, TNSF12, APO3L, CD255) is a member of the TNF superfamily of cytokines. TWEAK was first described in 1997 as a novel TNF-like protein displaying pro-apoptotic activity on interferon γ treated human HT-29 colon carcinoma cells [1] and since then has emerged as a prominent player in normal and pathological tissue remodeling. It is expressed as a 249 amino acid long type II membrane bound protein and comprises an intracellular N-terminal domain, which contains a potential protein kinase C phosphorylation site, a transmembrane domain and an extracellular TNF homology domain (THD). Upon specific proteolysis by the serine protease furin, soluble TWEAK is released from membranes [1]–[3].

Both membrane bound and soluble forms of TWEAK have been shown to be able to bind to the TWEAK receptor and trigger signaling [2]. The cognate TWEAK receptor (Fn14, TWEAKR, TNFRSF12A, CD266) is the smallest member of the TNF receptor superfamily (TNFRS) so far and interacts only with TWEAK [4]. The extracellular TWEAK binding domain of Fn14 comprises a single cysteine rich domain (CRD) that contains 3 disulfide bonds. It is structurally related to the CRD of other TNFRS members, some of which have been structurally characterized [5].

Signaling of Fn14 is initiated when TWEAK binds to the receptor and induces its trimerization. The signal is transmitted into the cell by attraction of the TNFR-associated factors (TRAFs) 1, 2, 3 and 5 to the short C-terminal cytoplasmic tail of Fn14 [6], [7]. This interaction leads to the activation of several signaling pathways including the ERK [8]–[10] and JNK [8], [11] pathways as well as the non-canonical [12]–[14] and canonical NF-κB signaling cascade [6], [7], [13]–[16]. The activation of the pathways results in the induction or repression of target gene expression.

Since its discovery, the TWEAK-Fn14 cytokine-receptor system has emerged from a weakly apoptosis-inducing signal to a key-player in the regulation of various, sometimes even opposing, cellular processes in tissue remodeling. Presence of TWEAK was shown *in vitro* to stimulate or inhibit proliferation, initiate or prohibit differentiation, support migration, prolong survival or induce cell death [1], [17]–[22]. Additionally, Maecker and colleagues revealed that TWEAK serves as a regulator of the innate immune system and its interplay with adaptive immunity. [23] The biological relevance of TWEAK is potentiated by the fact that in contrast to other TNF superfamily members TWEAK is a widely expressed cytokine in many different tissue and tumor specimens (for references see [24]). Its receptor Fn14 is expressed in all cell types analyzed so far, except primary B and T cells. In contrast to other TNFRS members, Fn14 expression is up-regulated by a wide range of cytokines, growth factors and Fn14 self-activation [8], [25], [26]. The large repertoire of cellular responses together with the broad range of cell types expressing TWEAK and Fn14 makes them key regulators of progenitor expansion, cell proliferation, cell migration, angiogenesis and inflammation during tissue repair after acute injuries and in physiological tissue remodeling [4], [8], [27]–[34].

All these processes have to be tightly regulated and any dissonance in this orchestra easily leads to pathological effects. Consequently, the TWEAK-Fn14 axis was shown to play a detrimental role in several diseases. The ambivalent nature of TWEAK signaling is reflected in its effects on tumors. On the one hand, TWEAK is indeed able to induce apoptosis in tumor cells [35]. On the other hand, Fn14 expression is up-regulated in many tumor cell lines and tumors promoting proliferation, angiogenesis, inflammation, cell invasion and metastasis [15], [17], [18], [26], [29], [36]–[41]. Imbalance in the regulation of TWEAK in the process of inflammation and immune modulation leads to the development of chronic inflammation and autoimmune diseases like rheumatoid arthritis [20], [42], systemic lupus erythematosus [43], [44], neuroinflammation [16], [45], multiple sclerosis [46], [47] and ischaemic stroke [48]–[51]. The involvement of the TWEAK-Fn14 axis in beneficial as well as hazardous processes, make both ligand and receptor potential targets for novel therapeutics.

Possible new therapeutic approaches based on monoclonal antibodies or antibody derivates can either directly block the TWEAK Fn14 interaction or kill the cells by targeting Fn14 with antibodies inducing Antibody-dependent cell-mediated cytotoxicity (ADCC), delivering toxins or triggering the intrinsic apoptotic potential of Fn14. However, no structural information of this important cytokine or its interaction with Fn14 is available, perhaps because TWEAK is a very sticky and difficult to handle protein and not well suited for forming highly ordered crystals. In addition, a complex of TWEAK with a potentially therapeutic antibody could help develop treatments aimed at neutralizing the activity of TWEAK in soluble form or in its membrane bound form. In this study we report the crystal structure of TWEAK in complex with a Fab derived from a humanized neutralizing anti-TWEAK rabbit antibody. We derive a molecular framework for TWEAK, differences and similarities to other TNF family cytokines and, by comparison with available structures of TNF superfamily members in complex with their receptors, our structure enables us to dock the Fn14 CRD to its putative binding site on the ligand TWEAK. In addition, the structure of human TWEAK suggests that binding to HSPGs is important for triggering signaling after acute injuries and supports tumor development as it is observed for other cytokines.

## Materials and Methods

### Half-Life Determination of the TWEAK-antibody Complex

A Biacore 2000 instrument was used with a Biacore SA and HBS-ET (10 mM HEPES pH 7.4, 150 mM NaCl, 1 mM EDTA, 0.05% Tween® 20). Biotinylated human soluble TWEAK was coupled to the chip at 150 RU. Antibodies (100 mM in HBS-ET) were injected at 100 nM with a flowrate of 100 µl/min for 2 min association time. The dissociation of the immune complex was monitored for 5 min at 25°C in HBS-ET. The kinetically rate limiting step of the complex dissociation phase in the interval [240 s–300 s] was taken to calculate the dissociation rate kd [1/s] (Biacore Evaluation Software 4.0). According to the equation t1/2 diss = ln(2)/(60×kd), the half-life of the immune complex in minutes was calculated.

### Crossblocking Experiment

A Biacore 3000 instrument was used at 25°C with a Biacore SA sensor and HBS-ET as system buffer (10 mM HEPES pH 7.4, 150 mM NaCl, 1 mM EDTA, 0.05% Tween® 20). The sensor was treated with EDC/NHS chemistry (Biacore) and blocked with ethanolamine to suppress unspecific binding. Finally, the sensor was conditioned with 3×1 min 1 M NaCl/50 mM NaOH and 1×1 min 10 mM HCl at 100 µl/min. The biotinylated TWEAK ligand was injected at 5 nM in HBS-ET at a flow rate of 30 µl/min over all flow cells. Subsequently the respective primary antibody was injected in a single flow cell at 10 µl/min for 4 min. The secondary antibody was injected over all flow cells with the same conditions as the primary antibody. The binding level of the primary and secondary antibody was monitored and the Molar Ratio (MR) was calculated as the quotient from the signal level of secondary antibody/primary antibody. The sensor was inactivated by a 1 min injection at 30 µl/min of 6 M guanidinium hydrochloride in 100 mM glycine buffer pH 1.5. The TWEAK ligand was inactivated. The baseline was stabilized by a 7 min injection of HBS-ET at 100 µl/min. Within 7 cycles, new biotinylated TWEAK ligand was captured in the flow cells at 294 RU +/−5 RU. As a control, 100 nM polyclonal sheep antibody (Roche) was injected instead of primary and secondary antibody.

### Neutralization of TWEAK-Fn14 Interaction

Blocking of TWEAK-Fn14 interaction was shown by receptor interaction ELISA. 96-well Maxisorp® plates (Nunc, Langenselbold, Germany) were coated with 100 µl 1 µg/ml human Fn14:Fc (extracellular domain of human Fn14 (amino acids 1–75) fused to Fc portion of human IgG1) in PBS per well for 1.5 h at room temperature and blocked with a solution of 5% FBS in PBS for 30 minutes at room temperature under shaking. In the meantime, human Flag-tagged soluble TWEAK (amino acids 106–249; 2.5 ng/ml in blocking solution) was incubated with different concentrations of anti-TWEAK antibody or hybridoma supernatant for 2 h at room temperature under shaking. After washing the Fn14-coated plate once with buffer (0.1% Tween® 20 in PBS), 100 µl of the TWEAK-antibody solution were transferred to each well and the plate was incubated for 1 h at room temperature, followed by four washes with wash buffer. Wells were filled with 100 µl of anti-FLAG-HRP detection antibody, diluted 1∶5000 in blocking buffer, and incubated for 1 h at room temperature. After four more wash steps, the signal was developed by addition of 100 µl 3,3,5,5-Tetramethylbenzidine (TMB) solution for approximately ten minutes. The reaction was stopped by adding 100 µl of 1 N 10 HCl, and absorbance measured at 450 nm (reference wavelength 620 nm).

### IL-8 Secretion ELISA

Blocking of TWEAK activity by the anti-TWEAK antibody in a cellular system was shown in an IL-8 secretion assay using A375 melanoma cells. 10,000 A375 cells (ATCC #CRL1619) were seeded per well of 96-well cell culture plate in 100 µl of growth medium (DMEM with 4.5 g/L glucose, with pyruvate and GlutaMAX™/10% FBS) and incubated at 37°C/5% CO2 for 48 h. Human recombinant soluble TWEAK was pre-incubated at 300 ng/ml with different concentrations of anti-TWEAK antibodies in growth medium for 30 minutes at room temperature. Then, 50 µl of the mixture were added to each well of the cell plate, followed by another 48 h-incubation to allow for IL-8 secretion. 20 µl of the cell supernatant were removed after centrifuging the plate for five minutes at 200×g and mixed with 980 µl of RD5P Calibrator Diluent from the “CXCL8 Quantikine ELISA” kit (R&D Systems). IL-8 was detected by the ELISA according to the manufacturer’s instructions.

### Protein Production and Crystallization

Chimeric rabbit anti-TWEAK monoclonal antibody was produced by standard procedures. A Fab fragment was prepared by papain digestion for 3 h. The Fc portion was removed using a HiTrap MabSelect Xtra column (GE Healthcare, Munich, Germany) and the Fab fragment purified by gelfiltration on a Sephadex 75 column (GE Healthcare, Munich, Germany) with 20 mM His-HCl, 140 mM NaCl, pH 6.0 as buffer. For complex formation of the Fab with human TWEAK, the Fab solution was used to directly dissolve freeze-dried recombinant human TWEAK (PeproTech GmbH, Hamburg, Germany) to a final molar ratio of 1∶1. To remove residual phosphate from the TWEAK preparation, the complex was desalted with 5 ml HiTrap Desalting columns (GE Healthcare, Munich, Germany) and 20 mM His-HCl, 140 mM NaCl, pH 6.0 as buffer. For crystallization, 1 µl protein solution (18 mg/ml) was mixed with 1 µl reservoir solution 30% w/v Ethanol/10% w/v PEG6000/100 mM Sodium Acetate. Crystals grew at 25°C using the hanging drop vapor diffusion method. Prior to flash freezing in liquid nitrogen, crystals were cryoprotected by adding 1 µl reservoir supplemented with 20% (v/v) 1,4-butanediol to the drop.

### Data Collection, Molecular Replacement and Structure Refinement

Diffraction Data were recorded at 100 K at the beamline X06SA (SLS/Switzerland) and processed with XDS [52] (Table 1). Initial phases were obtained by molecular replacement with PHASER [53] using the previously solved structure of a therapeutic antibody Fab fragment in complex with human TWEAK as search models (to be published). In total, one complex consisting of one Fab fragment and one TWEAK molecule could be positioned in the asymmetric unit. The complete biological relevant complex of a TWEAK trimer bound by three Fab fragments can be generated by applying three fold symmetry along the crystallographic axis. Initial models were completed and refined by iterative cycles of manual model building including water placement with COOT [54] and standard crystallographic refinement including positional refinement, bulk solvent correction, overall anisotropic B factor and TLS refinement with Phenix [55]. In the final refinement round of a twinned refinement with Phenix [56] using the twin law -k,-h,-l has been applied. Refinement and model statistics are shown in Table 2. Buried surfaces were calculated with AreaIMol [57]. Rigid body and positional refinement of the putative TWEAK-Fn14 complex was performed with CNS [58]. Structure figures were generated with PyMOL (www.pymol.org).

**Table 1:** Crystallographic data collection and model refinement statistics.

Data collection	
Beamline	X06SA (SLS)
Wavelength	0.933
Space group Unit Cell parameters	P3
a, b, c (Å)	101.64 101.64 57.59
α, β, γ (°)	90 90 120
Resolution (Å) (last shell)	30.0–2.5 (2.65–2.50)
Observed reflections (last shell)	84796 (13268)
Completeness (last shell)	99.5 (97.8)
Redundancy (last shell)	3.7 (3.7)
R_sym_ (last shell)	9.8 (15.8)
I/σ (last shell)	3.7 (3.7)
**Refinement**	
Resolution (Å)	30–2.5
No. reflections (test)	22932 (2294)
*R*_work_/*R*_free_	18.34/21.11
**No. atoms**	
Protein	4273
Water	100
**rmsd from ideal**	
Bond lengths (Å)	0.013
Bond angles (°)	1.625
**Ramachandran plot**	
Most favoured (%)	95.8
Additionally allowed (%)	4.02
Disallowed (%)	0.18
**PDB Accession Code**	4HT1

**Table 2:** Cross blocking assay.

Molar Ratio^1^ (%)	Antibody 2		
Antibody 1	chi TW-301	chi TW-304	chi TW-305
chi TW-301	0	6	9
chi TW-304	0	0	3
chi TW-305	0	1	0

1 The Molar Ratio (MR %) was calculated as the quotient of the secondary antibody binding signal to the primary antibody binding signal, both binding to the surface-presented TWEAK ligand.

## Results

### Structure Determination of the TWEAK-Fab Complex

Anti-human TWEAK antibodies were obtained by immunization of New Zealand white rabbits and selected for their ability to bind human TWEAK, neutralize TWEAK-Fn14 interaction and inhibit TWEAK-induced IL-8 secretion in A375 melanoma cells. From all binders, three showed inhibition of the TWEAK-Fn14 interaction with IC_50_ in the low nM range resulting in the reduction of IL-8 secretion with an IC_50_ around 700 nM (Table 3).

**Table 3:** Biochemical analysis of selected anti TWEAK antibodies.

	Inhibition of TWEAK-FN14 interaction	Half-life of immunocomplex	Inhibition of IL-8 secretion
	IC_50_ [ng/ml]	t/2 diss [min]	IC_50_ [ng/ml]
TW-301	3.4	n.d.	128
TW-304	2.8	n.d.	109
TW-305	2.5	n.d.	99
chi TW-301	2.8	110	121
chi TW-304	2.6	37	122
chi TW-305	2.6	147	104

To test whether the selected antibodies bind different epitopes we performed crossblocking experiments using surface plasmon resonance. The accessibility values of all tested antibodies are below 10%, which is in the noise of the assay. These data indicate that the tested antibodies bind to overlapping epitope regions (Table 2). For crystallization, the antibody forming the immune complex with the slowest k_off_ was chosen (TW305chi in Table 3). From this antibody, Fab fragments were produced and used for crystallization of a complex between human TWEAK and the Fab fragments.

**Figure 1:**
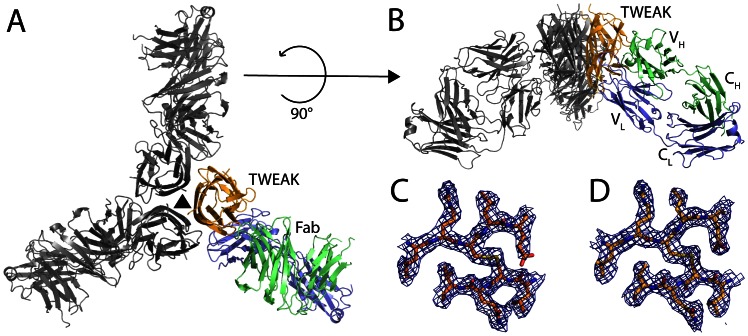
Overall structure of the TWEAK - Fab complex. A) Ribbon model of human TWEAK (orange) complexed with the Fab fragment of a therapeutic antibody (light chain in blue and heavy chain in green). The symmetry related two TWEAK – Fab complexes forming the physiological trimeric TWEAK are colored in gray with the corresponding 3-fold crystallographic axis depicted as a black triangle. The complex resembles a three-bladed propeller with each TWEAK-Fab protomer being one blade. B) Side view of the complex with same colors as in A. The Fab fragments are not binding planar but tilted ∼ 45° out of the plane to TWEAK leading to a trihedral shaped complex. C) Initial 2FoFc electron density after replacement as blue mesh around the disulfide bond of human TWEAK contoured at 1σ (amino acids as color coded sticks). D) Final 2FoFc electron density after refinement as blue mesh around the disulfide bond of human TWEAK contoured at 1σ (amino acids as color coded sticks).

The complex of soluble human TWEAK with the Fab fragment was reconstituted by directly dissolving lyophilized recombinant soluble human TWEAK in the buffer containing the Fab. In contrast to TWEAK alone, the reconstituted complex can easily be desalted without the reported problems of protein sticking to size exclusion matrices under low salt conditions [1]. The structure of the complex was solved by molecular replacement with a limiting resolution of 2.5 Å and refined to an R-factor of 23.8% (R_free_ = 28.4%). Examples of initial 2mF_o_-DF_c_ electron density after replacement and final 2mF_o_-DF_c_ density after coordinate refinement are shown in Figures 1C and D respectively. Complete crystallographic statistics are summarized in Table 1.

In total, one TWEAK molecule and one Fab fragment could be positioned in the asymmetric unit. Like other members of the TNF superfamily, soluble TWEAK is a homotrimer. This biological relevant trimer is found in the crystal and can be generated by applying the symmetry operation of the 3-fold crystallographic axis that coincides with the intrinsic 3-fold axis of the TWEAK trimer (Fig. 1A). The immune complex consists of one TWEAK homotrimer with three bound Fab fragments. When viewed along the 3-fold axis the hexameric complex has an overall shape that resembles a three-bladed propeller with each TWEAK-Fab protomer being one blade (Fig. 1A).

The pseudo 2-fold axis of the Fab fragments relating the heavy and light chains is not perpendicular to the 3-fold axis but the Fab fragments are tilted ∼ 45° out of the plane (Fig. 1B). The 3-dimensional arrangement of the hexameric complex resembles therefore a trigonal pyramid with the TWEAK trimer at the apex and the three Fab fragments pointing towards the 3 corners of the trigonal base (Fig. 1B). In this binding orientation the antibody would point away from the membrane in the situation of the membrane bound TWEAK precursor, with the membrane located above the TWEAK molecule in Figure 1B. Thereby the antibody not only binds soluble TWEAK, but in principle might be able to bind to TWEAK before it is released from its membrane bound precursor. On the one hand, the antibody can neutralize soluble TWEAK molecules. On the other hand, the antibody might be able to inhibit direct signaling of membrane bound TWEAK.

### Details of the TWEAK-Fab Interface

**Figure 2:**
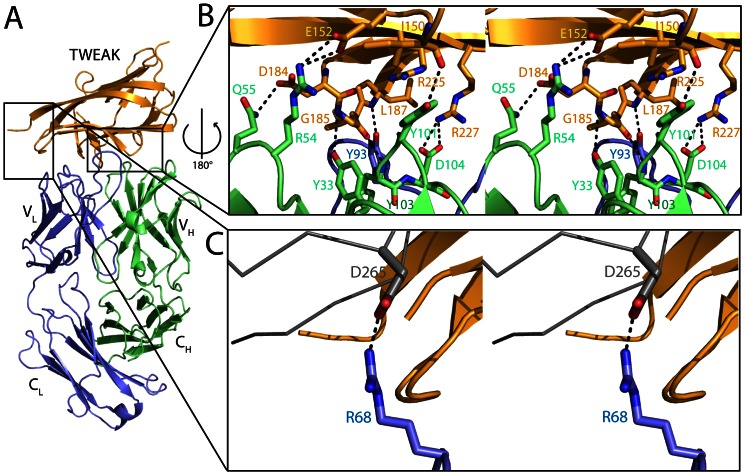
Interaction of the antibody with TWEAK. A) Ribbon representation of one Fab fragment binding to one TWEAK protomer (orange:TWEAK, blue:light chain, green:heavy chain). B) Stereo representation of the epitope recognition with interacting residues as labeled stick model and important hydrogen bond interaction highlighted as dashed lines. The binding is mainly mediated by CDR loop 1 and 2 of the heavy chain interacting with residues of the loops connecting strands D/E and B’/B and residues of strand G. In addition Y93 of CDR3 of the light chain interacts with a main chain N and stacks with the guanidinium group of R130 of TWEAK. C) Interestingly not only canonical CDR loops are involved in TWEAK binding, but an additional hydrogen bond is formed between light chain R68 of a non CDR loop with D75 of a second subunit of the trimeric TWEAK complex (gray).

**Figure 3:**
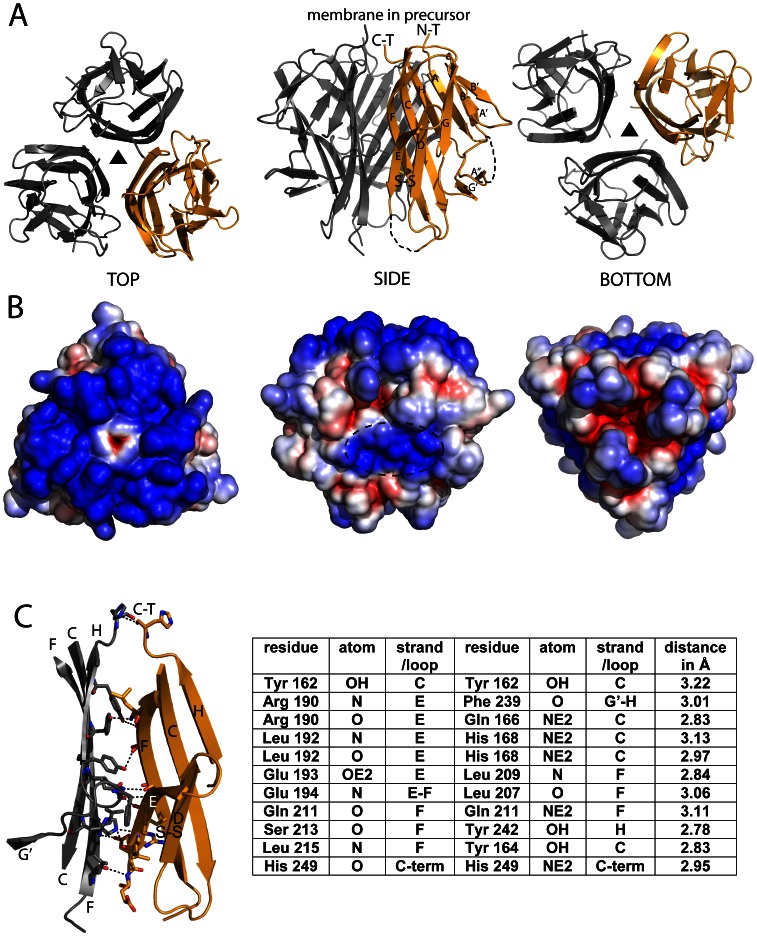
Structure of human TWEAK. A) Ribbon representations of the TWEAK trimer with one protomer colored orange and the symmetry related ones in gray (crystallographic 3-fold axis indicated as black triangle). On the left top view oriented as in 1A with N- and C-Terminus on the top. In the middle side view oriented as in 1B with labeled N- and C-Terminus. In the situation of the uncleaved precursor the membrane is located on top of the molecule. The disulfide bond is highlighted as stick model and beta strands are labeled according to TNF superfamily nomenclature. The dashed lines indicate flexible loops E-F and A-A’’ not visible in the electron density. On the right, bottom view of the TWEAK trimer. B) Solvent accessible electrostatic surface potential (red −4 kT to blue +4 kT) of the TWEAK trimer with the same orientations as in A. Resembling the high pI of TWEAK with 9.62 the complete upper surface is highly positively charged. A second basic patch is located at the side of the TWEAK trimer (dashed ellipse middle picture). This positively charged region is also found in other members of the TNF family (i.e. APRIL, BAFF) and coincides with their receptor binding site. C) Overview of the TWEAK-TWEAK interface as found in the homotrimer in the same orientation and labeled as in A (middle picture). Notable hydrogen bonds involved in the trimerization are indicated as dashed lines with the respective interacting amino acids as sticks. The hydrogen bonds with interacting atoms and distances are listed in the table.

The Fab binds to a non-linear epitope on human TWEAK. The recognition of the epitope is mainly achieved by the hypervariable region of the heavy chain. Notable hydrogen bonds involved in the binding are Y^H33^–G^T185^ (3.4 Å) of CDR H1, Q^H55^ - D^T184^ (3.1 Å) and R^H54^ - E^T152^ (3.3 Å) of CDR H2 and Y^H101^–I^T150^ (3.2 Å), Y^H103^–G^T185^ (2.7 Å), D^H104^–R^T227^ (2.8 Å) of CDR H3. From the light chain, a hydrogen bond is formed between Y^L93^ of CDR L3 and L^T187^ (2.7 Å) (Fig. 2C). In addition to the hydrogen bonds, a central π stacking interaction between Y^H101^ and R^T225^ supports the specificity towards the epitope (Fig. 2C). The residues of TWEAK forming the antigenic epitope are located in the loops connecting strands D/E and B’/B and residues of strand G (for numbering see Fig. 3C). The interaction of the Fab fragment with TWEAK buries in total an area of 882 Å^2^, which is in the typical range of the interaction surface between antibodies and protein antigens [59]. Besides this main antigen-antibody recognition, an additional interaction between residues R^L68^ located in a loop on one of the six canonical CDR loops and D^T265^ of a second TWEAK molecule of the TWEAK trimer is formed (Fig. 2C). However, both residues are located outside the core epitope and are solvent exposed. As a result of that exceptional position, the arginine can be mutated in the process of humanization to glycine without loss of affinity.

### Details of the TWEAK-TWEAK Interfaces

The biological relevant complex displays a 1∶1 stoichiometry. The epitope important for binding and specificity is located within a single TWEAK protomer and the Fabs do not bridge protomers in the trimer. The TWEAK protomer adopts the typical THD fold with a central sandwich of two five-stranded antiparallel β-strands with a smaller two-stranded one flanking them (Fig. 3A). The loops and strands harboring the residues of the antigenic epitope form part of one of the larger antiparallel β-sheets comprising strands B’, B, G, D and E. This sheet is facing the outside of the TWEAK trimer and is linked by a disulfide bond between residues C191 of strand E and C210 of strand F with the second five-stranded antiparallel β-sheet (Fig. 3A). The second sheet formed by strands A’, A, H, C and F is located inside mainly mediating the trimerization of TWEAK. The buried surface area between the TWEAK protomers is approximately 800 Å^2^, which is in agreement with the numbers for interactions of proteins of that size [60]. The interface spans almost the complete height of the molecule. Residues belonging to strands H, C, F and loop G’-H of one TWEAK protomer interact with residues of strands F and C of the corresponding sheet in the other protomer. Additional interactions are formed to strand E of the second large β-sheet as well as to loop E-F (Fig. 3C). In total, ten hydrogen bonds are formed in the core interface (listed in Fig. 3C). Together with some hydrophobic interactions these contacts lead to a tight self-trimerization of TWEAK. The trimeric state is important for the biological function as the trimerization of the Fn14 receptor upon TWEAK binding triggers the intracellular signaling. Since the signaling can be triggered by the membrane bound full length TWEAK [13], it is likely that the TWEAK precursor is already trimerized on the membrane.

### TWEAK Surface Properties and Model for Fn14 Interaction

**Figure 4:**
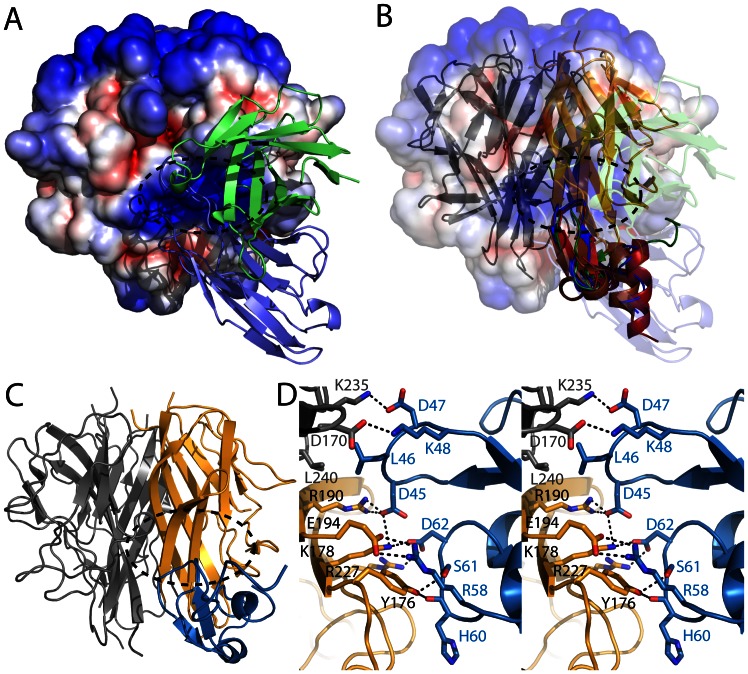
Model of the TWEAK – Fn14 receptor interaction. A) Side view of the TWEAK trimer showing the solvent accessible electrostatic surface potential (red −4 kT to blue +4 kT). The positively charged patch indicating the possible receptor binding site (dashed ellipse) is covered by the antibody selected for inhibiting TWEAK-Fn14 interaction (cartoon model of Hv in green and Lv in blue). B) Same view as in A with the antibody and TWEAK surface set transparence. After superposition of cytokine-receptor structures APRIL-BCMA (blue; PDB ID 1XU2), APRIL-TACI (brown; PDB ID 1XU1), TALL-BCMA (red; PDB ID 1OQD) and TALL-BAFFR (green; PDB ID 1OQE) the CRD of the receptors co-localize and mark the putative binding site of Fn14 on TWEAK (only the CRD of the receptors is shown as colored cartoon model). C) The NMR model of the Fn14 CRD (blue; PDB ID 2RPJ) is placed at the putative receptor binding site of TWEAK according to the complex structures shown in B. The basic patch is indicated with the dashed ellipse. Only one of the three receptors is shown. D) Stereo view of the modeled TWEAK-Fn14 CRD interface. Upon rigid body and positional refinement of the putative TWEAK-Fn14 CRD complex a dense hydrogen bond network is formed at the interface. The perfect complementarities of charged and hydrophobic patches, as well as the involvement of Fn14 side chains already shown to play an important role in TWEAK binding support this model.

To analyze surface properties of TWEAK, we calculated the electrostatic potential for the solvent accessible surface of the soluble TWEAK trimer. The surface potential reveals a basic patch located at the side of the TWEAK trimer (Fig. 3B middle and Fig. 4A). In our structure, this region is covered by the Fab fragment, which is derived from an antibody directed against disrupting the TWEAK-Fn14 interaction. Thus, it is likely that the basic patch is directly involved in the TWEAK-Fn14 interaction. To learn more about the interaction of TWEAK with its receptor, we first superimposed available structures of THDs in complex with the CRDs of their receptors, in particular APRIL (a proliferation inducing ligand) THD with BCMA (B cell maturation) CRD (PDB ID 1XU2), APRIL THD with TACI (Tumor necrosis factor receptor superfamily member 13B, also known as TNFRSF13B) CRD (PDB ID 1XU1), TALL THD with BCMA CRD (PDB ID 1OQD) and TALL CRD with BAFFR (receptor for B-cell activating factor) THD (PDB ID 1OQE)]. In fact, in all experimentally derived THD-CRD complexes, the receptors bind the cytokine at the position of the positive patch (Fig. 4B). Using this information, together with the NMR structure of human Fn14 CRD (PDB ID 2RPJ), we can generate a model for TWEAK-Fn14 by superimposing the human TWEAK structure with THDs and the NMR structure of Fn14 with the CRDs of the experimentally determined complexes. The model of the TWEAK-Fn14 complex is shown in Fig. 4C and can explain receptor trimerization without any observable clashes between the three Fn14 molecules.

To minimize energy of the complex model we used rigid body and positional refinement with CNS [58], which resulted in a highly complementary and specific binding interface between TWEAK and Fn14. The buried surface area of approximately 460 Å^2^ for the TWEAK-Fn14 interaction is smaller than for the other THD-CRD complexes. However, one can expect some rearrangements of the Fn14 molecule upon TWEAK binding compared to the present NMR structure in solution, which might lead to an increased interaction surface. Regardless of such rearrangements, the highly complementary interface, with approximately ten potential hydrogen bonds between the Fn14 CRD and TWEAK in the model, suggests a sufficiently strong receptor cytokine interaction, in agreement with the low experimental K_d_ values of ∼ 0.8–2.4 nM. In our model, the charged side chains of Fn14 residues D45, D47, K48, R58 and D62 recognize TWEAK and form the dense hydrogen bond network. This is in accordance with mutational analysis showing the importance of D45, K48 and D62 for TWEAK binding, but e.g. S38 and P56, which do not form direct interactions, being dispensable [61]. Thus, the model is consistent with and can nicely explain mutagenesis data.

### TWEAK Surface Properties and Model for HSPG Interaction

A second highly positively charged area spans the top of the TWEAK trimer (Fig. 3B left). A similar basic patch is observed for APRIL and other related molecules of the TNF family, indicating that this surface area is functionally important. In the case of APRIL, this basic patch is shown to bind to negatively charged sulphated side chains of HSPG. While binding of one soluble APRIL trimer to the receptor is not sufficient for signaling, the multimerization of APRIL upon interaction with the HSPGs and thereby the oligomerization of the receptors efficiently triggers signaling [62]–[66]. A similar observation was made for BAFF, TNF, CD95L, TRAIL and recently for TWEAK [13], [67]–[70]. Thus, consistent with these data, the positive patch on the TWEAK molecule is well suited and a likely surface area for a possible interaction with HSPGs.

## Discussion

Research over the past years established TWEAK as a multifunctional cytokine accompanying a key role in the various physiological processes especially tissue regeneration and reorganization [27]–[34]. Disregulation of the TWEAK-Fn14 cytokine receptor axis is involved in numerous pathological events including cancer (reviewed in [71]), chronic autoimmune diseases [20], [42]–[47] and acute ischaemic stroke [48]–[50], [72]. Accordingly, TWEAK is an interesting target in the development of antibody-based therapies against these diseases. Although several studies addressed the function of TWEAK and its receptor Fn14 in these processes *in vitro* and *in vivo,* no structural information of TWEAK or its interaction with Fn14 is available. In this study we report the crystal structure of the soluble form of human TWEAK in complex with the Fab fragment of a neutralizing antibody to obtain information on the structural features of TWEAK.

The complex formation with the Fab fragment was beneficial in several ways. The binding of the Fab fragment to human TWEAK greatly improved the handling of the protein. Whereas the free protein easily sticks to size exclusion matrices, the complex could easily be desalted and concentrated in complex with the Fab. In addition, neutralizing antibodies selected for the inhibition of ligand-receptor binding likely bind or block this interaction and can help determine the location of the receptor-ligand interface. Finally, the structure helps to develop antibody-based therapies against diseases where TWEAK is implicated. Comparison with available structures of other TNF superfamily members in complex with the CRD of their receptors revealed the putative receptor binding site on TWEAK, and this binding site is exploited and shielded by the Fab fragments. Based on this information we could also generate a model between human TWEAK and the Fn14 CRD that nicely explains and is supported by published mutational studies.

The interaction between TWEAK and its receptor is in contrast to many other TNF ligands and receptors unique [4]. This property makes the TWEAK-Fn14 axis as therapeutic targets especially interesting and potentially superior to other TNF family members. The occurrence of undesired side effects might be reduced compared to targeting ligands binding multiple receptors or receptors accepting different ligands. The presented structure shed light for the first time on the rather specific molecular recognition of TWEAK by Fn14 and provides a basis for the development and improvement of therapeutic molecules highly specific for targeting these two proteins. Our model provides a first glimpse into the specific interaction of TWEAK with Fn14. The superposition of the TWEAK structure and the NMR structure of the Fn14 onto available experimentally determined structures of related complexes resulted in a model that could be easily energy minimized to remove any residual clashes and possesses a highly complementary interface. In fact, the suggested specific hydrogen bonding and ion pair network is consistent with mutational analysis and explains the unique formation of the TWEAK-Fn14 complex. Furthermore, the model is consistent with Fn14 trimerization, which is an integral part of TWEAK mediated signaling.

Apart from TWEAK-mediated trimerization of Fn14, efficient signaling is only achieved by Fn14 oligomerization [73]. Receptor oligomerization is achieved either by membrane bound full length TWEAK or, in analogy to other TNF superfamily members, by binding of soluble TWEAK to HSPGs [13], [65]. In contrast to the specific interface between Fn14 and TWEAK, the identified basic patch on the opposite side of the receptor binding region of soluble TWEAK is likely to serve as a platform for binding to the negatively charged HSPG, via electrostatically driven interactions. Similarly, this positively charged surface is also found on related cytokines such as APRIL and hence suggests that HSPG-mediated stimulation of receptor oligomerization has related mechanisms in different THD-CRD complexes [63], [64].

In summary, we were able to obtain the first structural information of soluble human TWEAK. The crystallization was supported by the formation of a stable complex with the Fab fragment of a neutralizing antibody. Based on the comparison of the obtained TWEAK structure with published TNF ligand receptor structures, we were able to model a putative TWEAK-Fn14 complex. Furthermore, we identified a large positive surface patch that is likely to serve as a binding platform for TWEAK to negatively charged HSPG, thereby regulating the levels of TWEAK. Taken together our findings provide the profound molecular basis for future studies on the pleiotropic cytokine and potential drug target TWEAK and its receptor Fn14.

### Accession Number

Coordinates and structure factors for the crystal structure of human TWEAK in complex with a therapeutic antibody Fab fragment have been deposited in the Protein Data Bank with accession code 4HT1.

## References

[1] Chicheportiche Y, Bourdon PR, Xu H, Hsu YM, Scott H, et al (1997) TWEAK, a new secreted ligand in the tumor necrosis factor family that weakly induces apoptosis. J Biol Chem 272: 32401–32410. 9405449 10.1074/jbc.272.51.32401

[2] Brown SA, Ghosh A, Winkles JA Full-length, membrane-anchored TWEAK can function as a juxtacrine signaling molecule and activate the NF-kappaB pathway. J Biol Chem 285: 17432–17441. 10.1074/jbc.M110.131979 PMC2878507 20385556

[3] Marsters SA, Sheridan JP, Pitti RM, Brush J, Goddard A, et al (1998) Identification of a ligand for the death-domain-containing receptor Apo3. Curr Biol 8: 525–528. 9560343 10.1016/s0960-9822(98)70204-0

[4] Wiley SR, Cassiano L, Lofton T, Davis-Smith T, Winkles JA, et al (2001) A novel TNF receptor family member binds TWEAK and is implicated in angiogenesis. Immunity 15: 837–846. 11728344 10.1016/s1074-7613(01)00232-1

[5] He F, Dang W, Saito K, Watanabe S, Kobayashi N, et al (2009) Solution structure of the cysteine-rich domain in Fn14, a member of the tumor necrosis factor receptor superfamily. Protein Sci 18: 650–656. 19241374 10.1002/pro.49 PMC2760370

[6] Brown SA, Richards CM, Hanscom HN, Feng SL, Winkles JA (2003) The Fn14 cytoplasmic tail binds tumour-necrosis-factor-receptor-associated factors 1, 2, 3 and 5 and mediates nuclear factor-kappaB activation. Biochem J 371: 395–403. 12529173 10.1042/BJ20021730 PMC1223299

[7] Han S, Yoon K, Lee K, Kim K, Jang H, et al (2003) TNF-related weak inducer of apoptosis receptor, a TNF receptor superfamily member, activates NF-kappa B through TNF receptor-associated factors. Biochem Biophys Res Commun 305: 789–796. 12767899 10.1016/s0006-291x(03)00852-0

[8] Donohue PJ, Richards CM, Brown SA, Hanscom HN, Buschman J, et al (2003) TWEAK is an endothelial cell growth and chemotactic factor that also potentiates FGF-2 and VEGF-A mitogenic activity. Arterioscler Thromb Vasc Biol 23: 594–600. 12615668 10.1161/01.ATV.0000062883.93715.37

[9] Dogra C, Hall SL, Wedhas N, Linkhart TA, Kumar A (2007) Fibroblast growth factor inducible 14 (Fn14) is required for the expression of myogenic regulatory factors and differentiation of myoblasts into myotubes. Evidence for TWEAK-independent functions of Fn14 during myogenesis. J Biol Chem 282: 15000–15010. 17383968 10.1074/jbc.M608668200 PMC4149055

[10] Vendrell J, Maymo-Masip E, Tinahones F, Garcia-Espana A, Megia A, et al (2010) Tumor necrosis-like weak inducer of apoptosis as a proinflammatory cytokine in human adipocyte cells: up-regulation in severe obesity is mediated by inflammation but not hypoxia. J Clin Endocrinol Metab 95: 2983–2992. 20382683 10.1210/jc.2009-2481

[11] Wako M, Ohba T, Ando T, Arai Y, Koyama K, et al (2008) Mechanism of signal transduction in tumor necrosis factor-like weak inducer of apoptosis-induced matrix degradation by MMP-3 upregulation in disc tissues. Spine (Phila Pa 1976) 33: 2489–2494. 18923335 10.1097/BRS.0b013e318186b343

[12] Sanz AB, Sanchez-Nino MD, Izquierdo MC, Jakubowski A, Justo P, et al (2010) TWEAK activates the non-canonical NFkappaB pathway in murine renal tubular cells: modulation of CCL21. PLoS One 5: e8955. 20126461 10.1371/journal.pone.0008955 PMC2813291

[13] Roos C, Wicovsky A, Muller N, Salzmann S, Rosenthal T, et al (2010) Soluble and transmembrane TNF-like weak inducer of apoptosis differentially activate the classical and noncanonical NF-kappa B pathway. J Immunol 185: 1593–1605. 20610643 10.4049/jimmunol.0903555

[14] Saitoh T, Nakayama M, Nakano H, Yagita H, Yamamoto N, et al (2003) TWEAK induces NF-kappaB2 p100 processing and long lasting NF-kappaB activation. J Biol Chem 278: 36005–36012. 12840022 10.1074/jbc.M304266200

[15] Tran NL, McDonough WS, Savitch BA, Sawyer TF, Winkles JA, et al (2005) The tumor necrosis factor-like weak inducer of apoptosis (TWEAK)-fibroblast growth factor-inducible 14 (Fn14) signaling system regulates glioma cell survival via NFkappaB pathway activation and BCL-XL/BCL-W expression. J Biol Chem 280: 3483–3492. 15611130 10.1074/jbc.M409906200

[16] Polavarapu R, Gongora MC, Winkles JA, Yepes M (2005) Tumor necrosis factor-like weak inducer of apoptosis increases the permeability of the neurovascular unit through nuclear factor-kappa B pathway activation. J Neurosci 25: 10094–10100. 16267216 10.1523/JNEUROSCI.3382-05.2005 PMC6725778

[17] Kawakita T, Shiraki K, Yamanaka Y, Yamaguchi Y, Saitou Y, et al (2004) Functional expression of TWEAK in human hepatocellular carcinoma: possible implication in cell proliferation and tumor angiogenesis. Biochem Biophys Res Commun 318: 726–733. 15144899 10.1016/j.bbrc.2004.04.084

[18] Michaelson JS, Cho S, Browning B, Zheng TS, Lincecum JM, et al (2005) Tweak induces mammary epithelial branching morphogenesis. Oncogene 24: 2613–2624. 15735761 10.1038/sj.onc.1208208

[19] Harada N, Nakayama M, Nakano H, Fukuchi Y, Yagita H, et al (2002) Pro-inflammatory effect of TWEAK/Fn14 interaction on human umbilical vein endothelial cells. Biochem Biophys Res Commun 299: 488–493. 12445828 10.1016/s0006-291x(02)02670-0

[20] Perper SJ, Browning B, Burkly LC, Weng S, Gao C, et al (2006) TWEAK is a novel arthritogenic mediator. J Immunol 177: 2610–2620. 16888023 10.4049/jimmunol.177.4.2610

[21] Polek TC, Talpaz M, Darnay BG, Spivak-Kroizman T (2003) TWEAK mediates signal transduction and differentiation of RAW264.7 cells in the absence of Fn14/TweakR. Evidence for a second TWEAK receptor. J Biol Chem 278: 32317–32323. 12794080 10.1074/jbc.M302518200

[22] Felli N, Pedini F, Zeuner A, Petrucci E, Testa U, et al (2005) Multiple members of the TNF superfamily contribute to IFN-gamma-mediated inhibition of erythropoiesis. J Immunol 175: 1464–1472. 16034083 10.4049/jimmunol.175.3.1464

[23] Maecker H, Varfolomeev E, Kischkel F, Lawrence D, LeBlanc H, et al (2005) TWEAK attenuates the transition from innate to adaptive immunity. Cell 123: 931–944. 16325585 10.1016/j.cell.2005.09.022

[24] Burkly LC, Michaelson JS, Hahm K, Jakubowski A, Zheng TS (2007) TWEAKing tissue remodeling by a multifunctional cytokine: role of TWEAK/Fn14 pathway in health and disease. Cytokine 40: 1–16. 17981048 10.1016/j.cyto.2007.09.007

[25] Meighan-Mantha RL, Hsu DK, Guo Y, Brown SA, Feng SL, et al (1999) The mitogen-inducible Fn14 gene encodes a type I transmembrane protein that modulates fibroblast adhesion and migration. J Biol Chem 274: 33166–33176. 10551889 10.1074/jbc.274.46.33166

[26] Feng SL, Guo Y, Factor VM, Thorgeirsson SS, Bell DW, et al (2000) The Fn14 immediate-early response gene is induced during liver regeneration and highly expressed in both human and murine hepatocellular carcinomas. Am J Pathol 156: 1253–1261. 10751351 10.1016/S0002-9440(10)64996-6 PMC1876890

[27] Lynch CN, Wang YC, Lund JK, Chen YW, Leal JA, et al (1999) TWEAK induces angiogenesis and proliferation of endothelial cells. J Biol Chem 274: 8455–8459. 10085077 10.1074/jbc.274.13.8455

[28] Tanabe K, Bonilla I, Winkles JA, Strittmatter SM (2003) Fibroblast growth factor-inducible-14 is induced in axotomized neurons and promotes neurite outgrowth. J Neurosci 23: 9675–9686. 14573547 10.1523/JNEUROSCI.23-29-09675.2003 PMC6740475

[29] Jakubowski A, Ambrose C, Parr M, Lincecum JM, Wang MZ, et al (2005) TWEAK induces liver progenitor cell proliferation. J Clin Invest 115: 2330–2340. 16110324 10.1172/JCI23486 PMC1187931

[30] Girgenrath M, Weng S, Kostek CA, Browning B, Wang M, et al (2006) TWEAK, via its receptor Fn14, is a novel regulator of mesenchymal progenitor cells and skeletal muscle regeneration. EMBO J 25: 5826–5839. 17124496 10.1038/sj.emboj.7601441 PMC1698888

[31] Dohi T, Borodovsky A, Wu P, Shearstone JR, Kawashima R, et al (2009) TWEAK/Fn14 pathway: a nonredundant role in intestinal damage in mice through a TWEAK/intestinal epithelial cell axis. Gastroenterology 136: 912–923. 19109961 10.1053/j.gastro.2008.11.017

[32] Novoyatleva T, Diehl F, van Amerongen MJ, Patra C, Ferrazzi F, et al (2010) TWEAK is a positive regulator of cardiomyocyte proliferation. Cardiovasc Res 85: 681–690. 19887380 10.1093/cvr/cvp360

[33] Tirnitz-Parker JE, Viebahn CS, Jakubowski A, Klopcic BR, Olynyk JK, et al (2010) Tumor necrosis factor-like weak inducer of apoptosis is a mitogen for liver progenitor cells. Hepatology 52: 291–302. 20578156 10.1002/hep.23663

[34] Mittal A, Bhatnagar S, Kumar A, Paul PK, Kuang S (2010) Genetic ablation of TWEAK augments regeneration and post-injury growth of skeletal muscle in mice. Am J Pathol 177: 1732–1742. 20724600 10.2353/ajpath.2010.100335 PMC2947270

[35] Wang D, Fung JN, Tuo Y, Hu L, Chen C (2010) TWEAK/Fn14 promotes apoptosis of human endometrial cancer cells via caspase pathway. Cancer Lett 294: 91–100. 20189297 10.1016/j.canlet.2010.01.027

[36] Tran NL, McDonough WS, Donohue PJ, Winkles JA, Berens TJ, et al (2003) The human Fn14 receptor gene is up-regulated in migrating glioma cells in vitro and overexpressed in advanced glial tumors. Am J Pathol 162: 1313–1321. 12651623 10.1016/S0002-9440(10)63927-2 PMC1851233

[37] Ho DH, Vu H, Brown SA, Donohue PJ, Hanscom HN, et al (2004) Soluble tumor necrosis factor-like weak inducer of apoptosis overexpression in HEK293 cells promotes tumor growth and angiogenesis in athymic nude mice. Cancer Res 64: 8968–8972. 15604260 10.1158/0008-5472.CAN-04-1879

[38] Kawakita T, Shiraki K, Yamanaka Y, Yamaguchi Y, Saitou Y, et al (2005) Functional expression of TWEAK in human colonic adenocarcinoma cells. Int J Oncol 26: 87–93. 15586228

[39] Willis AL, Tran NL, Chatigny JM, Charlton N, Vu H, et al (2008) The fibroblast growth factor-inducible 14 receptor is highly expressed in HER2-positive breast tumors and regulates breast cancer cell invasive capacity. Mol Cancer Res 6: 725–734. 18505918 10.1158/1541-7786.MCR-08-0005 PMC3519279

[40] Dai L, Gu L, Ding C, Qiu L, Di W (2009) TWEAK promotes ovarian cancer cell metastasis via NF-kappaB pathway activation and VEGF expression. Cancer Lett 283: 159–167. 19398263 10.1016/j.canlet.2009.03.036

[41] Whitsett TG, Cheng E, Inge L, Asrani K, Jameson NM, et al (2012) Elevated expression of Fn14 in non-small cell lung cancer correlates with activated EGFR and promotes tumor cell migration and invasion. Am J Pathol 181: 111–120. 22634180 10.1016/j.ajpath.2012.03.026 PMC3388162

[42] Kamata K, Kamijo S, Nakajima A, Koyanagi A, Kurosawa H, et al (2006) Involvement of TNF-like weak inducer of apoptosis in the pathogenesis of collagen-induced arthritis. J Immunol 177: 6433–6439. 17056575 10.4049/jimmunol.177.9.6433

[43] Kaplan MJ, Lewis EE, Shelden EA, Somers E, Pavlic R, et al (2002) The apoptotic ligands TRAIL, TWEAK, and Fas ligand mediate monocyte death induced by autologous lupus T cells. J Immunol 169: 6020–6029. 12421989 10.4049/jimmunol.169.10.6020

[44] Leng RX, Pan HF, Qin WZ, Wang C, Chen LL, et al.. (2010) TWEAK as a target for therapy in systemic lupus erythematosus. Mol Biol Rep. 10.1007/s11033-010-0144-9 20358293

[45] Iocca HA, Plant SR, Wang Y, Runkel L, O’Connor BP, et al (2008) TNF superfamily member TWEAK exacerbates inflammation and demyelination in the cuprizone-induced model. J Neuroimmunol 194: 97–106. 18207576 10.1016/j.jneuroim.2007.12.003

[46] Desplat-Jego S, Feuillet L, Creidy R, Malikova I, Rance R, et al (2009) TWEAK is expressed at the cell surface of monocytes during multiple sclerosis. J Leukoc Biol 85: 132–135. 18945822 10.1189/jlb.0608347

[47] Serafini B, Magliozzi R, Rosicarelli B, Reynolds R, Zheng TS, et al (2008) Expression of TWEAK and its receptor Fn14 in the multiple sclerosis brain: implications for inflammatory tissue injury. J Neuropathol Exp Neurol 67: 1137–1148. 19018248 10.1097/NEN.0b013e31818dab90

[48] Potrovita I, Zhang W, Burkly L, Hahm K, Lincecum J, et al (2004) Tumor necrosis factor-like weak inducer of apoptosis-induced neurodegeneration. J Neurosci 24: 8237–8244. 15385607 10.1523/JNEUROSCI.1089-04.2004 PMC6729692

[49] Inta I, Frauenknecht K, Dorr H, Kohlhof P, Rabsilber T, et al (2008) Induction of the cytokine TWEAK and its receptor Fn14 in ischemic stroke. J Neurol Sci 275: 117–120. 18793781 10.1016/j.jns.2008.08.005

[50] Munoz-Garcia B, Moreno JA, Lopez-Franco O, Sanz AB, Martin-Ventura JL, et al (2009) Tumor necrosis factor-like weak inducer of apoptosis (TWEAK) enhances vascular and renal damage induced by hyperlipidemic diet in ApoE-knockout mice. Arterioscler Thromb Vasc Biol 29: 2061–2068. 19778942 10.1161/ATVBAHA.109.194852

[51] Haile WB, Echeverry R, Wu F, Guzman J, An J, et al (2010) Tumor necrosis factor-like weak inducer of apoptosis and fibroblast growth factor-inducible 14 mediate cerebral ischemia-induced poly(ADP-ribose) polymerase-1 activation and neuronal death. Neuroscience 171: 1256–1264. 20955770 10.1016/j.neuroscience.2010.10.029 PMC2991428

[52] Kabsch W (1993) Automatic processing of rotation diffraction data from crystals of initially unknown symmetry and cell constants. J Appl Cryst 21: 916–924.

[53] McCoy AJ, Grosse-Kunstleve RW, Adams PD, Winn MD, Storoni LC, et al (2007) Phaser crystallographic software. J Appl Crystallogr 40: 658–674. 19461840 10.1107/S0021889807021206 PMC2483472

[54] Emsley P, Cowtan K (2004) Coot: model-building tools for molecular graphics. Acta Crystallogr D Biol Crystallogr 60: 2126–2132. 15572765 10.1107/S0907444904019158

[55] Afonine PV, Grosse-Kunstleve RW, Adams PD (2005) A robust bulk-solvent correction and anisotropic scaling procedure. Acta Crystallogr D Biol Crystallogr 61: 850–855. 15983406 10.1107/S0907444905007894 PMC2808320

[56] Terwilliger TC, Grosse-Kunstleve RW, Afonine PV, Moriarty NW, Zwart PH, et al (2008) Iterative model building, structure refinement and density modification with the PHENIX AutoBuild wizard. Acta Crystallogr D Biol Crystallogr 64: 61–69. 18094468 10.1107/S090744490705024X PMC2394820

[57] Lee B, Richards FM (1971) The interpretation of protein structures: estimation of static accessibility. J Mol Biol 55: 379–400. 5551392 10.1016/0022-2836(71)90324-x

[58] Brunger AT (2007) Version 1.2 of the Crystallography and NMR system. Nat Protoc 2: 2728–2733. 18007608 10.1038/nprot.2007.406

[59] Davies DR, Cohen GH (1996) Interactions of protein antigens with antibodies. Proc Natl Acad Sci U S A 93: 7–12. 8552677 10.1073/pnas.93.1.7 PMC40169

[60] Jones S, Thornton JM (1996) Principles of protein-protein interactions. Proc Natl Acad Sci U S A 93: 13–20. 8552589 10.1073/pnas.93.1.13 PMC40170

[61] Brown SA, Hanscom HN, Vu H, Brew SA, Winkles JA (2006) TWEAK binding to the Fn14 cysteine-rich domain depends on charged residues located in both the A1 and D2 modules. Biochem J 397: 297–304. 16526941 10.1042/BJ20051362 PMC1513280

[62] Sakurai D, Hase H, Kanno Y, Kojima H, Okumura K, et al (2007) TACI regulates IgA production by APRIL in collaboration with HSPG. Blood 109: 2961–2967. 17119122 10.1182/blood-2006-08-041772

[63] Hendriks J, Planelles L, de Jong-Odding J, Hardenberg G, Pals ST, et al (2005) Heparan sulfate proteoglycan binding promotes APRIL-induced tumor cell proliferation. Cell Death Differ 12: 637–648. 15846369 10.1038/sj.cdd.4401647

[64] Ingold K, Zumsteg A, Tardivel A, Huard B, Steiner QG, et al (2005) Identification of proteoglycans as the APRIL-specific binding partners. J Exp Med 201: 1375–1383. 15851487 10.1084/jem.20042309 PMC2213192

[65] Kimberley FC, van Bostelen L, Cameron K, Hardenberg G, Marquart JA, et al (2009) The proteoglycan (heparan sulfate proteoglycan) binding domain of APRIL serves as a platform for ligand multimerization and cross-linking. FASEB J 23: 1584–1595. 19141538 10.1096/fj.08-124669

[66] Huard B, McKee T, Bosshard C, Durual S, Matthes T, et al (2008) APRIL secreted by neutrophils binds to heparan sulfate proteoglycans to create plasma cell niches in human mucosa. J Clin Invest 118: 2887–2895. 18618015 10.1172/JCI33760 PMC2447926

[67] Grell M, Douni E, Wajant H, Lohden M, Clauss M, et al (1995) The transmembrane form of tumor necrosis factor is the prime activating ligand of the 80 kDa tumor necrosis factor receptor. Cell 83: 793–802. 8521496 10.1016/0092-8674(95)90192-2

[68] Schneider P, Holler N, Bodmer JL, Hahne M, Frei K, et al (1998) Conversion of membrane-bound Fas(CD95) ligand to its soluble form is associated with downregulation of its proapoptotic activity and loss of liver toxicity. J Exp Med 187: 1205–1213. 9547332 10.1084/jem.187.8.1205 PMC2212219

[69] Wajant H, Moosmayer D, Wuest T, Bartke T, Gerlach E, et al (2001) Differential activation of TRAIL-R1 and -2 by soluble and membrane TRAIL allows selective surface antigen-directed activation of TRAIL-R2 by a soluble TRAIL derivative. Oncogene 20: 4101–4106. 11494138 10.1038/sj.onc.1204558

[70] Bossen C, Cachero TG, Tardivel A, Ingold K, Willen L, et al (2008) TACI, unlike BAFF-R, is solely activated by oligomeric BAFF and APRIL to support survival of activated B cells and plasmablasts. Blood 111: 1004–1012. 17942754 10.1182/blood-2007-09-110874

[71] Winkles JA, Tran NL, Berens ME (2006) TWEAK and Fn14: new molecular targets for cancer therapy? Cancer Lett 235: 11–17. 15885893 10.1016/j.canlet.2005.03.048

[72] Haile WB, Echeverry R, Wu F, Guzman J, An J, et al.. (2010) Tumor necrosis factor-like weak inducer of apoptosis and fibroblast growth factor-inducible 14 mediate cerebral ischemia-induced poly(ADP-ribose) polymerase-1 activation and neuronal death. Neuroscience. 10.1016/j.neuroscience.2010.10.029 PMC2991428 20955770

[73] Fick A, Lang I, Schafer V, Seher A, Trebing J, et al (2012) Studies of binding of tumor necrosis factor (TNF)-like weak inducer of apoptosis (TWEAK) to fibroblast growth factor inducible 14 (Fn14). J Biol Chem 287: 484–495. 22081603 10.1074/jbc.M111.287656 PMC3249102

